# Genome-wide CRISPR screens and their applications in infectious disease

**DOI:** 10.3389/fgeed.2023.1243731

**Published:** 2023-09-19

**Authors:** Kaveri Srivastava, Bhaswati Pandit

**Affiliations:** National Institute of Biomedical Genomics (NIBMG), Calcutta, West Bengal, India

**Keywords:** genome wide CRISPR screen, host pathogen interaction, infectious diseases, host factors, bacterial and viral disease

## Abstract

Inactivation or targeted disruption of a gene provides clues to assess the function of the gene in many cellular processes. Knockdown or knocking out a gene has been widely used for this purpose. However, recently CRISPR mediated genome editing has taken over the knockout/knockdown system with more precision. CRISPR technique has enabled us to perform targeted mutagenesis or genome editing to address questions in fundamental biology to biomedical research. Its application is wide in understanding the role of genes in the disease process, and response to therapy in cancer, metabolic disorders, or infectious disease. In this article, we have focused on infectious disease and how genome-wide CRISPR screens have enabled us to identify host factors involved in the process of infection. Understanding the biology of the host-pathogen interaction is of immense importance in planning host-directed therapy to improve better management of the disease. Genome-wide CRISPR screens provide strong mechanistic ways to identify the host dependency factors involved in various infections. We presented insights into genome-wide CRISPR screens conducted in the context of infectious diseases both viral and bacterial that led to better understanding of host-pathogen interactions and immune networks. We have discussed the advancement of knowledge pertaining to influenza virus, different hepatitis viruses, HIV, most recent SARS CoV2 and few more. Among bacterial diseases, we have focused on infection with life threatening *Mycobacteria*, *Salmonella*, *S*. *aureus*, etc. It appears that the CRISPR technique can be applied universally to multiple infectious disease models to unravel the role of known or novel host factors.

## Introduction

Nucleotide change at predetermined positions or genome editing are important tools in molecular biology and medicine. Targeted integration or deletion of part of the genome disrupts the loci which aids in understanding the linkage between a phenotype and gene to uncover the underlying function of a gene (Adli, 2018). This is usually followed by the application of selection pressure which results in screening of the phenotype of interest. Using this approach and its variations, the genetic basis of many fundamental processes like cell cycle control (Hartwell et al., 1970; Nurse, 1975), embryogenesis (Nüsslein-Volhard and Wieschaus, 1980), and cell death (Ellis and Horvitz, 1986) has been uncovered. Silencing or knocking out a gene enables us to understand the role of the gene in the cellular environment. RNAi technology has been widely used to disrupt gene function. It utilizes RNA molecules for suppression of the gene by the formation of double-stranded RNA involving either blocking of transcription or translation leading to the silencing of the gene (Yu and Yusa, 2019). However, a major caveat of this technology is the error prone off-target activity and a high propensity of false positives.

Given these drawbacks and continuous search for better methods led to the discovery of zinc finger nucleases (ZFNs). It utilizes the FokI endonuclease domain fused with an array of specific DNA-binding domain leading to double-strand breaks in the DNA, causing site-specific mutagenesis (Urnov et al., 2010). Designing the ZFNs was time-consuming and challenging with difficulties in delivery and stringent requirements to design the ZFNs (Yu and Yusa, 2019). Later, transcription activator-like effector nucleases (TALENs) were used for site-specific mutagenesis (Baker, 2012). This modular system is based on TAL effector DNA binding proteins, fused with FokI endonuclease. TALENs were significantly larger in size which made delivery of TALEN machinery difficult to the cells (Gupta and Musunuru, 2014).

The advent of clustered regularly interspersed short palindromic repeats (CRISPR) and CRISPR-associated protein 9 (Cas9) technologies have revolutionized the way to interrogate gene function and has increasingly displaced RNAi/ZFN/TALENs as the method to generate targeted genome-wide mutagenesis in biomedical research (Doudna and Charpentier, 2014). CRISPR is part of the immune system in bacteria against phage infections (Barrangou et al., 2007). In genome engineering, the Cas9 enzyme causes double-strand breaks when guided by guide RNA (gRNA) which has sequence homology to the locus (Jinek et al., 2012). gRNA or sgRNA (single guide RNA) is comprised of 20-22 bp long sequence crRNA fused to scaffold tracrRNA. Cas9 enzyme has polymerase, nuclease, and helicase activity. Through sequence homology, gRNA guides Cas9 to specified genetic loci next to the protospacer adjacent motif (PAM). The CRISPR/Cas9 complex binds to DNA and cleaves it. The host machinery takes over and repairs the double-strand breaks through Non-Homologous End Joining (NHEJ), which disrupts the gene expression and function (Jinek et al., 2012).

The specificity and enhanced efficiency of Cas9 endonuclease to target specific genes can be achieved by changing the sequence of the gRNA. This has led to the generation of genome-wide CRISPR knockout (KO) libraries (Sanjana et al., 2014; Shalem et al., 2015) and genome-wide CRISPRi libraries (Sanson et al., 2018). These optimized libraries are designed in a way that they equally represent all the genes and perform across all gRNAs. Libraries are usually tagged with a selective marker, mostly antibiotic resistance or fluorescence. CRISPR libraries can be used to knock out, inhibit or activate target genes, CRISPR knockouts being the most common one (Bock et al., 2022). The libraries combine specific sgRNAs with Cas9 or Cas9 derivatives. Typically, CRISPR-based screens are carried out by using lentivirus to deliver a “pooled” gRNA library to a mammalian Cas9-expressing cell line. Following transduction with the gRNA library, mutant cells are screened for a phenotype of interest (e.g., survival, drug resistance, growth) to identify novel genes that modify the desired phenotype (Shalem et al., 2015). Cas9 derivatives are engineered Cas9 protein derived from bacteria. For example, catalytically dead Cas9 (dCas9) lacks the endonuclease activity, which means it cannot cleave DNA. Instead, it can be used for gene repression (CRISPRi) by being fused with repressor domains to inhibit gene expression. dCas9 has been used in identification of processes which regulate promoter activity, epigenetic modifications, visualization of genomic loci in living cells and more (Komor et al., 2016).

The genome-wide CRISPR screens enable mapping of genetic interactions because of their programmability and ease of use. This technique applies selective pressure on the population of cells with genes knocked out leading to the identification of genes that are either enriched or depleted in the selected cell population relative to the control population (Bock et al., 2022). Use of an unbiased sgRNA library provides an extremely powerful way to identify novel protein functions as knocking out disrupts the whole cellular environment. Distinguishing and identification of the mutants within the pooled population of cells plays a major role in the deconvolution of genetic interactions. The direct linkage between gene disruption and change in phenotype is the key to CRISPR screens (Yu and Yusa, 2019). However, CRISPR mediated screens have few limitations. Most knockout libraries contain gRNAs that target unique regions of constitutively expressed 5′exons. Targeting such 5′exons increases the likelihood that frameshift mutations will result in complete loss of protein expression (Doench et al., 2014). Moreover, mutations induced by Cas9 may maintain the open-reading frame, resulting in minor insertions or deletions in the final protein. In this scenario, the “knockout” effect will depend on how essential the exon to which gRNA is designed is to protein function. It also does not guarantee a functional knockout, which may result in false-negative results (Shalem et al., 2014).

**FIGURE 1:**
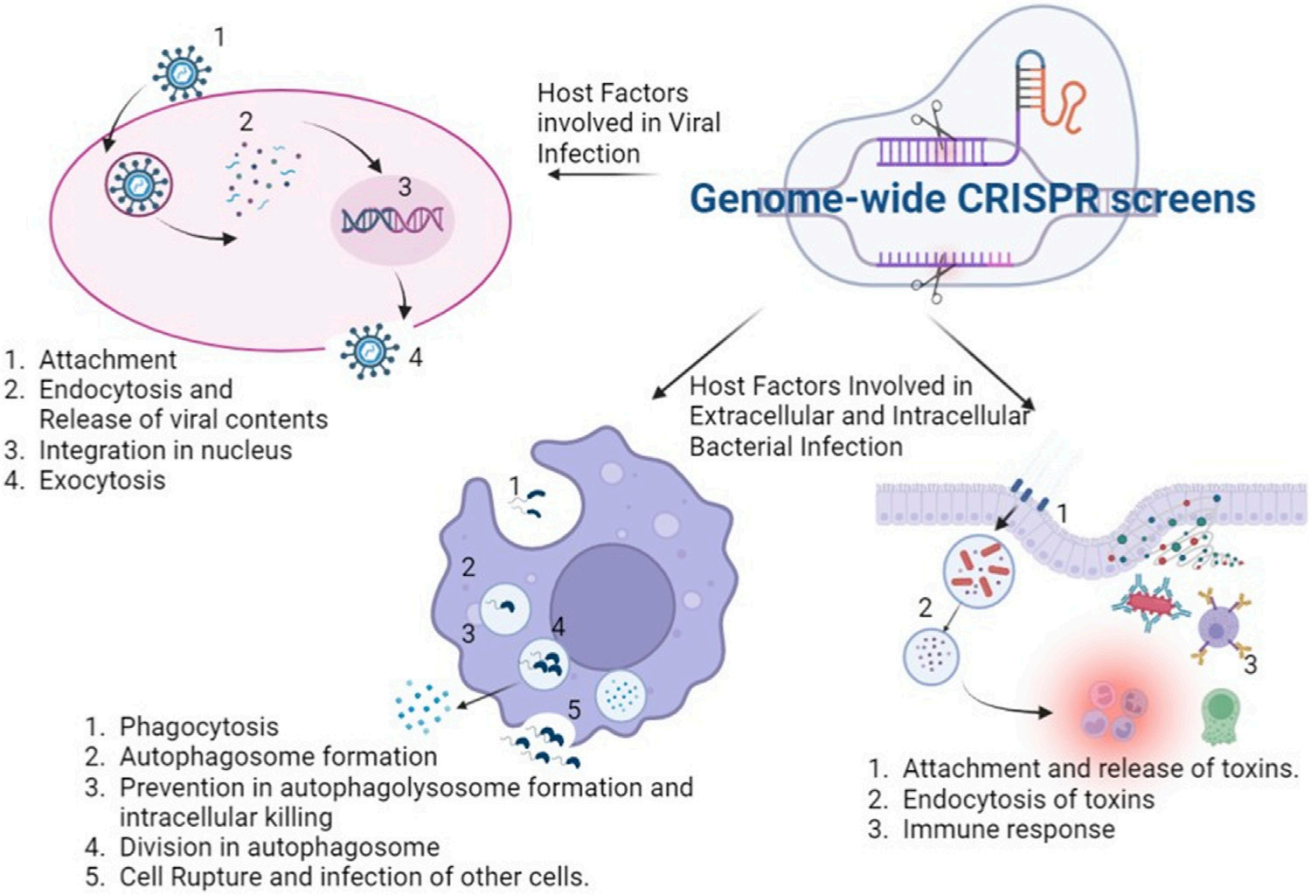
Application of genome wide CRISPR screens in various infectious diseases: the genome wide CRISPR screens can discover host factors which aid in viral attachment, entry into cell, release of viral content and integration into nucleus and lastly formation and release of viral particles. It can unravel host factors facilitating bacterial survival inside and outside the host cells and in the cross talk with the pathogen. The figure was created with BioRender.com.

Recent times have seen repeated emergence of various pathogens from HIV to SARS-CoV2 along with the presence of widespread diseases like tuberculosis, polio, smallpox, diphtheria, having substantial morbidity and mortality. Discovery of host factors involved in disease progression as well as pathogenesis are required for host-directed therapies. Genome-wide CRISPR screens provide a strong mechanistic way to discover these host factors and host dependency factors which can be further targeted in therapeutic ways (Figure 1). In this review article, we have attempted to compile the studies describing infectious disease where genome wide CRISPR screens have been applied to discover host factors implicated in host-pathogen interactions as well as understanding of pathogenesis (Table 1).

**TABLE 1:** Comprehensive summary of the studies described in the review.

Pathogen	Cell type	Library	Key genes involved in infection	References
Influenza	A549	GeCKO v2	SLC35A1, IGDCC4 and ZFAT	Song et al. (2021)
	A549	AVANA4	WDR7, CCDC115, TMEM199, and CMTR1	Li et al. (2020)
	A549	GeCKO v2	SLC35A1, GDF11, IRX3, C2CD4C, TRIM23, PIGN, ACADSB, GRAMD2, CIC, JAK2 and PIAS3	(Han et al., 2018)^a^
	HEK-293SF	Brunello	DDX6, SMG9, CARM1, SLC35A1, DPH1	(Sharon et al., 2020)^a^
	A549	GeCKO v2	SLC35A1, CMAS, GNE, NANS, CYTH2, ABCD2	(Yi et al., 2022)^a^
HIV	GXRCas9	Custom Library	TPST2, SLC35B2, and ALCAM	Park et al. (2017)
	J-LAT A2	Library designed using sequences from Sabatini/Lander CRISPR pooled library	KDM1B, KDM4A, KDM5A, MINA53, UTY	Huang et al. (2019)
	Jurkat T cell line	GeCKO v2	ARL-16, ATF1, CGREF1, USMG5, and ZNF304	Krasnopolsky et al. (2020)
Flaviviruses	iPSCs	Library designed using sequences from Sabatini/Lander CRISPR pooled library	TM9SF2, ATP6V1C1, ATP6V1F, SSR2, SSR3, EMC2, EMC6, ISG15, SOCS3, STAT3	Li et al. (2019)^a^
	Glioblastoma stem cells	Brunello	CENPH, ITGB5, MYLPF, HOMER1, BAALC, GABBR2, EPHA10, PTPN2, GCNT7, TRAM1, and TMEM41B	Wang et al. (2020)
	HuH7.5.1	GeCKO v2	STT3A, STT3B, MAGT1, OST4, RPN2	Lin et al. (2017)^a^
	HuH7.5.1	GeCKO v2	SSR1, SSR2 and SSR3, STT3A, STT3B, RPN2, OSTC, VMP1, RPS25	Marceau et al. (2016)^a^
	HuH7.5.1	GeCKO v2	EMC1, EMC5, EMC6, BAX, RACK1	Shue et al. (2021)^a^
	HAP1	GeCKO v2	TMEM41B, VMP1, TMEM64, ATG13, ATG14	Hoffmann et al. (2021)^a^
	HAP1	GeCKO v2	SEC61A1, SSR3, STT3A, STT3B, RPN2, DPM1, DPM3	Labeau et al. (2020)^a^
	HeLa	GeCKO v2	RAB5C, RABGEF1, WDR7, ZFYVE20, SSR2, SSR3, STT3A, EXT1, EXTL3	Savidis et al. (2016)^a^
	293FT	Custom Library	SEL1L, UBE2J1, EMC3, EMC2, DERL2, UBE2G2, HRD1	Ma et al. (2015)^a^
	293FT	GeCKO v2	STT3A, SEC63, SEC61B, OSTC, SPCS1, SPCS3, SERP1, EMC6, SEL1L, HSPA13, OST4, EMC4	Zhang et al. (2016)^a^
Chronic hepatitis B	HepG2	Library designed using sequences from Sabatini/Lander CRISPR pooled library	ZCCHC14, TENT4A, TENT4B, PARP10	Hyrina et al. (2019)
Hepatitis A virus	Huh7.5.1	GeCKO v2	GNE, CMAS, SLC35A1, UGCG, ST3GAL5, EIF4B, EIF3C, EIF3CLI, UFM1, UBA5, UFL1, UFC1, UFPS2, PAPD5/7, ZCCHC14	Kulsuptrakul et al. (2021)
Hepatitis C virus	HuH7.5.1	GeCKO v2	CD81, OCLN, OCLN1, PPIA, ELAVL1, CSKNK1A1, DNMT1	Marceau et al. (2016)
	HuH7.5.1	Custom Library	CD81, OCLN, PPIA, TRIM26	Liang et al. (2021)^a^
Herpesviruses	SVEC4-10 endothelial cells	GeCKO v2	EXTL1-3, SLC35B2, B3GAT3, Nrp-1	Lane et al. (2020)
Sindbis virus	HCT116	Brunello	SLC35B2, B4GALT7, EXT1, EXT2, COG3, COG4, and COG8	Petitjean et al. (2020)
SFTSV	HeLa	GeCKO v2	SNX11, KRIT1, AHR, SRRD, ZBTB6, KRTAP5-3, PTPN11	Liu et al. (2019)
SARS-CoV2	HuH7.5.1	GeCKO v2	TMEM41B, MINAR1, FKBP8	Kratzel et al. (2021)
	A549	GeCKO v2	ATP6AP1, ATP6V1A, NPC1, RAB7A, CCDC22, and PIK3C3	Daniloski et al. (2021)
	Vero	*C*. *sabaeus* genome-wide pooled CRISPR library	Cathepsin L, ACE2, DPP4, HMGB1, ARID1A, SMARCB1, SMARCC1	Wei et al. (2021)
	HuH7.5.1	GeCKO v2	TMEM106B, VAC14, SCAP, MBTPS2, EXOC2	Wang et al. (2021)
	HuH7.5.1	Brunello	PIK3C3, TMEM41B, EXT1, ITGB6, OSBPL9, PTDSS1, TMEM30A, CCZ1B, PTPN23, RSG1	Baggen et al. (2021)^a^
	HEK293T	Human Genome-wide CRISPRa-v2 Libraries	TMEM30A, CTSL, CPLX1, LDLRAD3, GPM6B, EPHB1, CLEC4G, MASP1, CLEC5A, HLA-DQA1, ICAM2, STAMBPL1	Zhu et al. (2021)
*S*. *aureus*	U937 macrophages	GeCKO v2	ADAM10, SYS1, ARFRP1, TSPAN14 and SGMS1	Winter et al. (2016)
*Mycobacterium tuberculosis*	THP1 macrophages	Brunello and Dolcetto	TYK2, IFNAR2, IFNAR1, STAT2, JAK1, IRF, ARNT, AHR, MAPK14, CHUK, HDAC2	Lai et al. (2020)
	RAW264.7	GeCKO v2	IFNAR1, IFNAR2, JAK1, STAT1, STAT2	Zhang et al. (2021)^a^
*Salmonella sps*	THP1 macrophages	GeCKO v2	ACTR3, ARPC4, CAPZB, CYFIP2, TOR3A, CTTN, CLTCL1, HMGCR, ATPA2, B3GNT1, PDGF, NHLRC2	Yeung et al. (2019)
*Legionella pneumophila*	U937 macrophages	Library designed by Morgen et al.	C1orf43, KIAA1109, RAB10, RABIF, SAR1A, SEC13, SEC23B	Jeng et al. (2019)
Enterohemorrhagic *Escherichia coli*	HT-29	AVANA	A4GALT, B4GALT5, UGCG, TM9SF2, LAPTM4A	Pacheco et al. (2018)

^a^ Overlapping host factors from different studies.

## Application of genome-wide CRISPR screens in understanding molecular basis of infectious diseases

### Viral diseases

Viruses enter the host cell and take control of the cell’s internal machinery to produce additional virus particles. It is always fascinating to observe the mechanisms by which virus particles enter host cells, hijack the host’s cellular machinery for their own benefit, and suppress the host’s immune system. Genome-wide CRISPR technique is able to provide answers to these queries with new directions and identify novel genes as well. Each of the following studies described below have addressed these vital issues and provided comprehensive knowledge on mechanisms in different viral diseases. Many studies have also validated earlier findings from siRNA-based screening.

The Influenza virus known to cause acute respiratory infection. CRISPR screens performed on lung epithelial cell A549 infected with highly pathogenic H5N1 strain of influenza virus for 2 days using GeCKO library identified *SLC35A1*, *IGDCC4* and *ZFAT* genes necessary for viral replication of which *IGDCC4* is a novel one (Song et al., 2021). Knocking out the *IGDCC4* (transmembrane protein immunoglobulin superfamily DCC subclass member 4) gene reduced viral replication significantly suggesting its role in the early stage of viral replication. IGDCC4 interacts with viral hemagglutinin through its extracellular domain and facilitates internalization of the virus into the host cells not only for influenza A but also other subtypes of influenza viruses such as H1N1 virus (A/WSN/1933) and H9N2 virus (A/chicken/Jiangsu/C4258/2012). However, *IGDCC4* deficient A549 cells did not take up transferrin or Vesicular Stomatitis Virus suggesting that IGDCC4 mediated endocytosis is specific for influenza virus. The role of IGDCC4 in virus replication and internalization was also validated *in vivo* in a mouse model infected with H5N1 (Song et al., 2021). A new approach, Meta-Analysis by Information Content (MAIC) was devised to systematically combine results obtained from the screens with prior evidence for influenza host factors (Li et al., 2020). This screen validated host derived factors obtained from previous screens and novel factors like *TRAPP* and *TEEX2* complex, genes essential in prenylation, co-factors of V type ATPase. The study also demonstrated that knocking out of *WDR7*, *CCDC115*, and *TMEM199* protected both A549 cells and human lung fibroblasts from infection with laboratory strains PR8 as well as clinical strains H1N1 and H5N1. All these genes mentioned above are essential for viral entry (not attachment), regulation of V-type ATPase assembly and endosomal pH maintenance. Incoming virions were prevented from nuclear entry and retained in the endolysosomal compartment due to impaired viral fusion in the knockout cells. It was observed that *CMTR1*, a human mRNA cap methyltransferase, is required for efficient viral cap snatching demonstrated by elF4E antibody pulldown assay for capped viral and host RNA. *CMTR1* also helps in the regulation of cell’s autonomous immune response and provides synergistic protection with the influenza endonuclease inhibitor Xofluza (Li et al., 2020). Development of MAIC to compile data from all genetic and proteomics screens are useful resources to identify the therapeutic targets. Another independent study on A549 cells infected with H5N1 infection revealed many host dependency factors. The top hit genes are members of vacuolar ATPase family, proton transport, and vacuolar acidification. Besides, sialic acid biosynthesis, protein glycan modification, GPI anchor synthesis pathway were also enriched which consists of host genes like *SLC35A1*, *GDF11*, *IRX3*, *C2CD4C*, *TRIM23*, *PIGN*, *ACADSB*, *GRAMD2*, *CIC*, *JAK2*, and *PIAS3*. Disruption of all of these genes significantly reduced viral replication. When beta lactamase carrying influenza virus-like particles were used to measure the ability of clonal knockouts to support virion entry and/or fusion, only knocking out *SLC35A1* restricted the entry and fusion of influenza A virus in the cells. Knocking out *CIC* (capicua) which is a conserved DNA binding transcriptional repressor, heightened antiviral state rendering cells less permissive to viral replication suggesting CIC as a negative regulator of cell’s intrinsic immunity (Han et al., 2018). Additionally, a genome wide CRISPR screen incorporating a reporter virus and FACS selection to identify and rank factors that restrict the entry of influenza virus, validated the findings from all the above-mentioned screens. All cellular factors that restrict viral production can be considered as potential candidates for production of vaccines in future (Sharon et al., 2020). The validation study reiterates the fact that Genome-wide CRISPR screens are highly reproducible across different laboratories when experimental conditions are the same.

Lastly, another genome wide CRISPR screen using GeCKOv2 library also identified different set of host factors *NANS*, *CYTH2*, *MIIP*, *TTC24*, *NXPH2*, *HLA-G* and *STI8* in addition to previously discovered factors like *SLC35A1*, *CMAS*, *GNE* and *SLC35A2*. Knocking out these genes led to reduction in viral load in A549 cells, suggesting that these genes play key role in H7N9 virus infection (Yi et al., 2022). Knocking out *SLC35A1*, *CMAS*, *GNE* and *NANS* suppressed binding of virus to the cells, whereas knocking out *CYTH2* and *ABCD2* reduced viral entry. CYTH2 is a GEF that replaces ARF1/3/6 GDP with GTP and is involved in vesicular transport whereas ABCD2 is an ATP binding cassette. Together they may function in virus internalisation and transportation to cytoplasm. Knocking out of *SLC35A2*, *MIIP* along with other genes lowered viral polymerase activity. *CYTH2* deficient cells treated with SecinH3, a small antagonist of cytohesin GEF, reduced LDH release but did not affect cell viability after H7N9 infection. This suggests that *CYTH2* suppresses H7N9 replication in a GEF dependent manner. Fusion of viral membrane and endosomal membrane was blocked in *CYTH2* knockout cells and can be used as target for vaccine production to treat Influenza virus infection (Yi et al., 2022).

Extensive screening using RNAi identified many host derived factors associated with HIV although few were consistent across multiple studies (König et al., 2007; Brass et al., 2008). However, genome-wide CRISPR screens discovered novel host factors involved in entry and transmission in engineered physiologically relevant T cell line, GXRCas9 after infection with CCR5-tropic HIV-1 strain JR-CSF (Park et al., 2017). The screen identified *CD4* and *CCR5*, known HIV co-receptors along with three additional genes, Tyrosylprotein sulfotransferase 2 (*TPST2*), Solute carrier family 35 member B2 (*SLC35B2*), and Activated leukocyte cell adhesion molecule (*ALCAM*). Knocking out *TPST2* and *SLC35B2* conferred resistance to infection which was reverted back upon rescue with introduction of sgRNA resistant cDNA of these genes. Additionally, TPST2 and SLC35B2 are involved in sulfation of proteins in trans Golgi. Earlier it was demonstrated that sulfation of CCR5 in trans Golgi is important for interaction with HIVgp120. Lack of sulfation in *TPST2* and *SLC35B2* knockout cells prevented entry of viruses despite comparable protein expression in wild type (GXRCas9) and knockout cells. This study reiterates the importance of sulfation to facilitate the recognition of HIV envelope by the CCR5 receptor. In addition, the *ALCAM* null cell did not exhibit abnormalities in terms of proliferation and virus infection but grew as a single cell preventing cellular aggregation characteristics of primary T cells. ALCAM mediated cell-cell aggregation is necessary in HIV transmission between cells which was disrupted when knocked out by sgRNA (Park et al., 2017). Rescue experiments with addition of cDNA, coculturing with WT cells or transmission of HIV virions validated the necessity of cell contact in effective HIV transmission. Importantly, these findings were validated in activated primary T cells isolated from healthy donors highlighting the significance of so-called non-essential host proteins in HIV pathogenesis. Combination antiretroviral therapy has significantly reduced HIV mortality and co-morbidity, but challenges exist pertaining to latent HIV in CD4^+^ T cells. Comprehensive screening has identified two major pathways, namely, histone modifications and SWI/SNF complex as critical host factors in HIV latency in J-LAT A2 cells using genome wide CRISPR knockout library developed by Sabatini and Lander (Wang et al., 2014). Targeting histone demethylases (*KDM1B*, *KDM4A*, *KDM5A*, *MINA53*, *UTY*) led to reactivation of HIV. Particularly, a novel host factor, *MINA53* a jumonji domain containing histone demethylase was found to increase H3K36me3 and H4K16Ac at 5′LTR of HIV proviruses. The absence of *MINA53* in J-LAT-A2 cells resulted in elevated levels of H3K36 methylation facilitating recruitment of KAT8, a known mediator of H4K16Ac at the HIV promoter. Inhibition of MINA53 using pan jumonji demethylase inhibitor JIB-04 reversed latency and activated the dormant HIV viruses (Huang et al., 2019). Similarly, another study identified several genes *ARL-16*, *ATF1*, *CGREF1*, *USMG5* and *ZNF304* in maintaining HIV latency in HIV-infected Jurkat T cell line using GeCKOv2 library. ChIP-qPCR experiments validated elevated occupancy of ZNF304 at the HIV promoter. However, in *ZNF304* knockout cells, there was overall HIV gene transcription which could be reversed on ectopic expression of *ZNF304*. *ZNF304* deficient cells exhibited moderately reduced enrichment of H3K9me3. TRIM28 recruits SETB1 to HIV promoter to mediate H3K9 methylation through ZNFs. Hence knocking out *ZNF304* led to low occupancy of TRIM28 and SETB1 on HIV promoter (Krasnopolsky et al., 2020). Thus, genome wide CRISPR screens have enriched the knowledge on mechanisms of viral entry, latency and epigenetic modifications.

The genera flavivirus constitutes many enveloped single-stranded positive sense RNA viruses. Some of the medically important viruses belong to this genus, like Dengue (DENV), Japanese Encephalitis (JEV), West Nile (WNV), Zika (ZIKV), and Yellow Fever (YFV) viruses. DENV is an arthropod-borne virus causing febrile disease, which in extreme conditions could cause hemorrhagic fever (Bhatt et al., 2013). ZIKV is known to cause congenital abnormalities like microcephaly and other birth defects in the fetus (Fitzgerald et al., 2018). JEV causes disease in children and WNV mostly affects birds but can affect humans when there is a spillover (Mulvey et al., 2021). YFV causes fever, nausea and body ache and in severe conditions could affect the liver leading to jaundice (Garske et al., 2014).

Flaviviruses infection have conserved cycle consisting of the following steps, viral entry and attachment via receptor mediated endocytosis, release of viral RNA after fusion with endosomal membrane, genome replication and translation in Endoplasmic Reticulum (ER), virion packaging and release through secretory pathway of trans-Golgi and release of viruses by exocytosis (Kanojia et al., 2022). The genome wide CRISPR screens have identified host factors in each of these steps which aid in viral pathogenesis. The screens were performed in various cell types like HuH7.5.1 (Marceau et al., 2016; Lin et al., 2017; Shue et al., 2021), HAP1 cells (Labeau et al., 2020; Hoffmann et al., 2021), HeLa cells (Savidis et al., 2016), glioblastoma stem cells (Wang et al., 2020), 293FT cells (Ma et al., 2015; Zhang et al., 2016) and iPSCs (Li et al., 2019) using different genome wide CRISPR libraries available commercially like GeCKO v2 (Marceau et al., 2016; Savidis et al., 2016; Zhang et al., 2016; Lin et al., 2017; Labeau et al., 2020; Hoffmann et al., 2021; Shue et al., 2021), Brunello (Wang et al., 2020), Sabatini and Lander genome wide CRISPR library (Li et al., 2019) or by designing own library using CustomArray (Ma et al., 2015). *Tyro3*, *Axl*, and *Mer* (TAM) family receptor tyrosine kinases along with *NDST1* and *EXT1* genes are involved in heparan sulfation necessary for virus attachment. All these proteins interact with envelope proteins of ZIKV, DENV, WNV (Savidis et al., 2016). Additionally, integrin αvβ5 aids internalisation of ZIKV in glioblastoma stem cells. Blocking of integrin αvβ5 using antibodies or inhibitors led to reduction in ZIKV infection (Wang et al., 2020). Host Signal Peptidase Complex (SPCS) participates in cleavage of flavivirus proteins (prM and E). *SPCS1*, a significant component of SPCS, when knocked out, reduced the replication of flaviviruses along with reduced cleavage of prM and E proteins (Zhang et al., 2016). Screens revealed host signal recognition particle (SRP)- translocon pathway proteins SEC61A1 and SEC63 aids in integration of viral polypeptide into the ER of the host interfering with maturation and assembly of virus particles (Marceau et al., 2016; Zhang et al., 2016). Furthermore, independent studies discovered components of Endoplasmic Reticulum protein Complex (EMC) as important host factors (Ma et al., 2015; Marceau et al., 2016; Savidis et al., 2016; Hoffmann et al., 2021). EMC helps in stabilization and insertion of viral protein to ER membrane. Besides, ER resident dolichol-phosphate mannose synthase (DPMS) complex was identified as host dependency factor for DENV and ZIKV. DPMS helps in synthesis of dolichol phosphate mannose which donates mannose to proteins in ER. This leads to N-glycosylation, glycosylphosphatidylinositol anchor biosynthesis, and C- or O- mannosylation of proteins. Such post translational reaction helps in host aided proper glycosylation and folding of viral structural proteins (Labeau et al., 2020). The translated viral proteins required for viral RNA synthesis assemble in the ER along with host ER proteins. Host proteins STT3A and STT3B components of Oligosaccharyltransferases (OST) complex, help in co-translational glycosylation of glycoproteins and acceptor sites required for DENV replication but only STT3A is required for ZIKV replication. Knocking out *STT3A* led to reduction in replication of other viruses like YFV, WNV and JEV. All of these defects were rescued, when a catalytically dead mutant of *STT3A* was expressed. This suggests that the presence of the OST complex is necessary for viral RNA replication (Marceau et al., 2016). Similarly, another study identified *MAGT1*, a different component of OST complex with oxidoreductase activity as an essential host factor in DENV propagation. It was observed that STT4B regulates MAGT1 expression and MAGT1 is associated with NS1 and NS4B proteins of DENV (Lin et al., 2017). All these studies provide new insights into the role of proteins which interact with each other influencing viral replication.

CRISPR screen identified multiple host factors of diverse function. Transmembrane Protein 41B (*TMEM41B*), a host protein actively participate in formation of replication complex at the ER membrane together with viral proteins NS4A and NS4B (Hoffmann et al., 2021). The receptor for Activated Kinase 1 (RACK1), a host protein, is known for its function in protein anchoring, shuttling, and stabilization. Besides, RACK1 also mediates specific cellular pathways through protein-protein interactions. A CRISPR screen in HuH7.5.1 established RACK1 to play an important role in the replication of viral RNA in DENV, ZIKV, and WNV but not in YFV by interacting with NS1 of the flaviviruses (Shue et al., 2021). Identification of host proteins with Golgi function and interferon activity imparting resistance to ZIKV infection in human pluripotent stem cell-derived neural progenitor cells (Li et al., 2019) were also resultant of CRISPR screens.

Chronic hepatitis B (CHB) virus infection leads to enhanced risk of severe liver diseases, including cirrhosis and hepatocellular carcinoma (Trépo et al., 2014). The key characteristic of this infection is presence of high levels of non-infectious small lipid HBV surface antigen (HBsAg) in circulation which is reduced with treatment. RG7834 targets the terminal nucleotidyltransferase proteins 4A and 4B (TENT4A and TENT4B or PAPD5 and PAPD7) thereby inhibiting expression of HBsAg (Zoulim and Durantel, 2015). To identify host factors involved in HBsAg expression and elucidation of molecular mechanism of action of RG7834, genome-wide CRISPR screen was performed on HepG2 cells harbouring an integrated form of HBV genome using library designed by Cellecta (Hoffman et al., 2014). The most enriched factor promoting production of HBsAg was a zinc finger CCHC-type containing 14 (ZCCHC14) protein, along with TENT4A/B, stabilizing HBsAg expression through HBV RNA tailing, providing a potential new therapeutic target to achieve functional cure in CHB patients (Hyrina et al., 2019).

A genome-wide CRISPR screen has provided insights into the host cellular factors involved in replication of the positive-sense RNA virus, Hepatitis A virus (HAV) known to cause acute inflammation in the liver (Matheny and Kingery, 2012). The Huh7.5.1 cells were infected with the cytopathic HAV HM175/18f strain and GeCKOv2 library was used to perform the screen. The top hits of the screen are involved in sialic acid/ganglioside biosynthesis (*GNE*, *CMAS*, *SLC35A1*, *UGCG*, *ST3GAL5*) and components of eukaryotic translation initiation factor complex (*EIF4B*, *EIF3C*, *EIF3CLI*), indicating their putative role in IRES mediated translation of HAV. Additionally, an intriguing pathway, UFMylation (*UFM1*, *UBA5*, *UFL1*, *UFC1*, *UFPS2*), not previously described in HAV infection was found to be enriched. UFMylation was shown to be necessary for viral translation through UFM1 conjugation of host ribosomal protein RPL26 and components related to the TRAMP complex independent of poly(A) tails or RNA stability. Furthermore, the screen identified two non-canonical polyA RNA polymerases, PAPD5 and PAPD7 as hits. PAPD5/7 complexes with ZCCHC14 to form a TRAMP-like complex. Double knockout of PAPD5/7 resulted in significant reduction in viral RNA. RG7834 an inhibitor of PAPD5/7 affects viral RNA in the same manner as in double knockout. Inhibition of TRAMP led to decreased HAV replication demonstrated in liver hepatocytes and in liver organoid cultures suggesting its potential as a target for host-directed therapies against HAV infection (Kulsuptrakul et al., 2021). Interestingly, the discovery of common protein ZCCHC14 along with PAPD5/7 was made in studies on HAV and Chronic HBV infection suggesting that these viruses share similar pathways for their survival within the host.

Genome wide CRISPR screens have identified multiple host derived factors (*CD81*, *OCLN*, *OCLN1*, *PPIA*, *ELAVL1*, *CSKNK1A1*, *DNMT1*) required for HCV replication and survival in Huh7.5.1 cells using GeCKOv2 library. In hepatocytes, receptors CD81, occludin, and claudin1 are found to be cardinal for the entry of Hepatitis C virus (Marceau et al., 2016). During an independent screening conducted on HuH7.5.1 infected with HCV, TRIM26, an E3 ligase, was discovered to be a significant host factor in the infection of Hepatitis C virus. Deficiency of *TRIM26* impaired replication of HCV but not of ZIKV or DENV. Functional studies demonstrated that host TRIM26 interacts with HCV-encoded viral polymerase NS5B protein, and thus mediating K27-linked ubiquitination leading to enhanced interaction of NS5B with NS5A (Liang et al., 2021). However, mouse TRIM26 cannot interact with NS5B due to its extra six amino acid insertions. The interaction was restored by deletion of the extra amino acids. Thus, *TRIM26* plays a vital role in viral host tropism allowing HCV to replicate in selective species.

A wide range of infections is caused by herpesviruses. High throughput screens discovered several host factors necessary for transcription and translation of β-herpesvirus murine cytomegalovirus (MCMV) infection in SVEC4-10 endothelial cells using GeCKOv2 library. Top hit genes are involved in heparan sulphate synthesis like *EXTL1-3*, *SLC35B2*, *B3GAT3* and COG family genes. Novel host cell factors required for infection were found to be Vascular endothelial growth factor (VEGF) and semaphorin-binding receptor Neuropilin-1 (Nrp-1). Knocking out *Nrp-1* showed early viral gene expression and reduced infection rates in endothelial cells, fibroblasts, and macrophages. The cells deficient of *Nrp-1* were more susceptible to TNF mediated necroptosis. Blocking Nrp-1 using anti-Nrp-1 antibodies reduced the infection drastically, suggesting the importance of Nrp1 in establishing MCMV infection (Lane et al., 2020).

Sindbis virus, an enveloped double stranded RNA virus from the Togaviridae family, cause sindbis fever which is present with maculopapular exanthema (Kurkela et al., 2004). Genome wide CRISPR screen has identified contribution of heparan sulphate pathway as a crucial factor for dsRNA entry, sensing and apoptosis in HCT116 human cells when challenged with either SINV or synthetic dsRNA using Brunello library. Heparan sulphate may serve as a receptor for viral entry. Enriched genes in screens *SLC35B2*, *B4GALT7*, *EXT1*, and *EXT2* are members of heparan sulfate pathway and *COG3*, *COG4*, and *COG8* are part of conserved oligomeric Golgi complex. Additionally, in this study, a novel function of COG4, a constituent of the conserved oligomeric Golgi (COG) complex, was revealed as a critical factor in promoting survival of SINV in human cells. Reduced viral production as well as reduced viral dsRNA transfection efficiency were observed when *COG4* was knocked out. This was due to decreased extracellular heparan sulfate which led to increased cell survival of *COG4* knockout cells (Petitjean et al., 2020). Absence of *SLC35B2*, *B4GALT7* also led to reduced dsRNA transfectability, SINV infectivity and infection induced cell death.

Severe fever with thrombocytopenia syndrome virus (SFTSV) is known to cause lethal infections and febrile thrombocytopenia syndrome (SFTS) in humans (Yu et al., 2011). Genome wide CRISPR screen discovered an essential host factor, sorting nexin 11 (*SNX11*) in HeLa cells using GeCKO v2 library for SFTSV infection. Knocking out *SNX11* prompted reduction in viral RNA suggesting SNX11’s role in establishment of SFSTV infection. SFSTV colocalized with the markers for early endosomes (RAB5a), late endosomes and endolysosomes (RAB7a), and lysosomes (LAMP1). The glycoproteins of SFTSV were identified and localised in later endosomal compartments but could not be recognized in the endoplasmic reticulum (ER) or Golgi mechanical assembly of *SNX11* deficient cells. All these observations are suggestive of involvement of SNX11 in intracellular endosomal trafficking pathway. SNX11 was found to regulate the pH of endosomes, facilitating viral replication. In comparison to regular HeLa cells, the endosomal compartments of *SNX11* deficient cells exhibited higher pH values and colocalized with V-ATPase. Additionally, higher expression of lysosomal-associated membrane protein 1 (LAMP1) indicated the involvement of SNX11 in maintaining the pH of endosomes, which aids in viral replication (Liu et al., 2019). LAMP1 and SNX11 both can be potential host targets to treat SFSTV infection.

The current situation has perked up the research in the context of infection of SARS-CoV2. SARS-CoV2 has shown increased infectivity and comparatively low fatality leading to widespread transmission and global pandemic. Genome-wide CRISPR screens have been conducted to uncover various key players in host-pathogen interactions. The studies conducted on SARS-CoV 2 infection have provided insights into novel receptors that aid in viral entry, proteases which process viral proteins and replication. To investigate the hypothesis that evolutionarily distinct viruses of the corona family share the same pathway to establish its infection, studies were carried out in GeCKO sgRNA library containing HuH7 cells infected with MERS-CoV and SARS-CoV2. Notably the study revealed genes involved in autophagy like *TMEM41B*, *MINAR1*, and the immunophilin *FKBP8* as common host factors required for pan-CoV replication. The findings were validated in the human nasal epithelial cell model by inhibiting the immunophilin protein family with the clinically approved drugs cyclosporine A, and the non-immunosuppressive derivative alisporivir (Kratzel et al., 2021). Additionally, genome wide CRISPR screens performed on human alveolar epithelial cells A549 using GeCKOv2 with infection of SARS-CoV2 identified hits clustered into distinct pathways including the vacuolar ATPase proton pump, Retromer, and Commander complexes (Daniloski et al., 2021). Similarly, screens in Vero-E6 cells infected with SARS-CoV2, Middle East respiratory syndrome CoV (MERS-CoV), bat CoV HKU5 expressing the SARS-CoV1 spike, and vesicular stomatitis virus (VSV) expressing the SARS-CoV2 spike using a *C*. *sabaeus* genome-wide pooled CRISPR library highlighted host factors, including the receptor ACE2 and protease Cathepsin L. This study identified pro-viral genes like *HMGB1* and important pathways, like SWI/SNF chromatin remodelling complexes playing a significant role in viral entry. HMGB1 regulates *ACE2* expression and is critical for the entry of SARS-CoV2, SARS-CoV1, and NL63 (Wei et al., 2021). The study using GeCKOv2 library also highlighted phosphatidylinositol phosphate biosynthesis and cholesterol homeostasis as essential host pathways that are crucial in facilitating infection by all three coronaviruses in HuH7.5.1 cells (Wang et al., 2021). In contrast, the lysosomal protein TMEM106B appeared unique to SARS-CoV2 infection as it enhanced production of more viruses using Brunello library mediated screening was performed in HuH7 cells (Baggen et al., 2021). Study led by Zhu et al. identified tissue specific novel receptors other than ACE2 which aid viral entry to the respective tissues in HEK293T cells using CRISPRa library (Zhu et al., 2021). LDLRAD3 expressed in neurons serve as an entry point for the SARS-CoV-2 into neurons while CLEC4G, a glycan-binding receptor and member of the C-type lectin family mediate SARS-CoV-2 entry and infection into liver cells, lymph nodes and monocytes (Zhu et al., 2021). LDLRAD3 also serves as a critical receptor for the Venezuelan equine encephalitis virus (VEEV) entry into the brain (Ma et al., 2020). The non-ACE2 receptor binds to the NTD region of spike protein rather than the RBD domain. These factors can be considered as the target for host-directed therapies against SARS-CoV-2 infection.

### Bacterial diseases

Bacterial infections are caused due to proliferation of harmful bacteria inside the human body and can be lethal in case of highly pathogenic bacterial infection. Bacterial infections are controlled with the help of antibiotics. Bacteria commonly affecting hosts are *Staphylococcus aureus*, *E*. *coli*, *Klebsiella pneumoniae*, *Mycobacterium tuberculosis*, *Salmonella*, etc.

Alpha hemolysin (αHL) is a toxin released by *S*. *aureus*, oligomerizes and forms pore on plasma membrane (Berube and Bubeck Wardenburg, 2013). To identify the host factors involved in alpha hemolysin (αHL) toxicity, genome-wide CRISPR screen was performed using GeCKO v2 library (Winter et al., 2016). Human myeloid U937 cells transduced with GeCKOv2 lentivirus treated with *S*. *aureus* derived αHL for 2 weeks, identified the receptor gene *ADAM10* as a top hit that can bind to the toxin. Additionally, other genes *SYS1*, *ARFRP1*, *TSPAN14* and *SGMS1* were important. Systematic disruption of all of these genes led to resistance to αHL toxin. However, susceptibility to αHL was restored by complementation of each of these genes. Knocking out these genes impaired binding of the toxin to the cell surface. SYS1, ARFRP1, TSPAN14 regulate expression of ADAM10 on cell surface. The specificity of loss of ADAM10 towards αHL binding was also proven by unchanged sensitivity to streptolysin O (SLO), another pore forming toxin of group A, C, G *Streptococcus*. Interestingly, deficiency of sphingomyelin synthase 1 (SGMS1) exhibited resistance to αHL intoxication but was more susceptible to SLO. Similarly, an independent study using mutagenized human haploid cells also identified ADAM10 and PLEKHA7 necessary for αHL toxicity. All these investigations improved our understanding of the molecular pathogenesis of *S*. *aureus* infection (Winter et al., 2016).

Tuberculosis is an infectious disease caused by *Mycobacterium tuberculosis (M*.*tb)* with an annual death toll of 1.2 million globally (WHO, 2018). Type I interferon (Lai et al., 2020; Zhang et al., 2021) and aryl hydrocarbon receptor (AHR) signalling (Lai et al., 2020) pathways were highlighted to be important host factors from two independent studies using genome-wide CRISPR screens reported. Lai et al. used Brunello library to perform CRISPR KO screen and Dolcetto library to perform CRISPRi screens in THP1 macrophages after 3 rounds of infection with BCG (Lai et al., 2020). Zhang et al. used GeCKOv2 library to perform screens in RAW264.7 macrophages infected with *M*.*tb* H37Rv (Zhang et al., 2021). Other host factors enriched in the both the screens were *TYK2*, *IFNAR2*, *IFNAR1*, *STAT2*, *JAK1*, *IRF*, *ARNT*, *AHR*, *MAPK14*, *CHUK*, *HDAC2* of which few were known earlier. Blocking type I interferon led to augmentation of the rifampicin effectiveness in *M*.*tb-*infected mice (Zhang et al., 2021). Use of cerdulatinib, a small molecular inhibitor for the type I interferon pathway and mAb for IFNAR1 led to better survival of human macrophages and clearance of intracellular *M*.*tb*. The *M*.*tb* inhibits apoptosis in infected macrophages, but induces type 1 interferon mediated cell death that facilitates the dissemination of the *M*.*tb* to uninfected macrophages (Zhang et al., 2021). Similar phenomenon was observed when the aryl hydrocarbon receptor signalling pathway was blocked with the help of CH223191 (Lai et al., 2020) establishing the role of AHR. The knowledge of different activated pathways at the molecular level may contribute to designing of host-directed therapies more systematically.

*Salmonella sps*. are known to cause various enteric diseases in humans. The genome-wide CRISPR screen (GeCKOv2) performed on THP1 macrophages infected with GFP tagged *Salmonella* identified 183 genes involved in actin dynamics, glycosaminoglycan metabolism, receptor signalling, lipid raft formation, calcium transport, and cholesterol metabolism (Yeung et al., 2019). All these genes form well defined first order protein-protein networks implicating their importance, however relevance of some pathways in host pathogen interaction were poorly understood (actin dynamics). Interestingly some of the key findings from this study overlap with findings from another study on identification of genes involved in phagocytic uptake (Haney et al., 2018). Top 19 genes were selected for validation through single gene knockout. In all cases the host cells with disrupted genes were resistant to *Salmonella* infection. Binding with chemical inhibitors identified a novel gene NHLRC2 to play a vital role in *Salmonella* infection. NHLRC2 is well known regulator in actin cytoskeleton regulation thereby connecting the two phenomena (Yeung et al., 2019). NHLRC2 mutants were strongly resistant to *Salmonella* possibly through downregulation of receptors involved in pathogen recognition which facilitate *Salmonella* uptake. NHLRC2 mutant cells also display unusual morphology not suitable for bacterial uptake. More detailed studies are necessary to confirm these findings.

A severe form of pneumonia called Legionnaires’ disease is caused by *Legionella pneumophila*. Genome wide CRISPR screens using lentiviral library described by Morgens et al. in U937 macrophages revealed C1orf43 and KIAA1109 as regulators of phagocytosis to engulf the bacterium (Morgens et al., 2017). It was observed that RAB10, a key GTPase and its chaperone RABIF are required for optimal *L*. *pneumophila* replication and ER recruitment of the *Legionella*-containing vacuole. Additionally, ER and Golgi genes involved in COPII vesicle biogenesis (*SAR1A*, *SEC13*, *SEC23B*) were also enriched in the screen suggesting importance of COPII mediated transport of secretory proteins from ER. Deletion of these genes prevented the *Legionella* mediated killing of macrophages. There were many more (*EXOC1-3*, *EXOC5-8*) novel hits discovered from this comprehensive study which help us for better understanding of *Legionella* infection (Jeng et al., 2019).

The foodborne human pathogen Enterohemorrhagic *Escherichia coli* (EHEC) is known to cause diarrheal illness worldwide. There are two pivotal factors responsible for its virulence: a type III secretion system (T3SS) and Shiga toxins (Stxs). Genome wide CRISPR screens was conducted in HT-29 colonic epithelium cells using AVANA library and infecting with ΔespZ derivative of EHEC strain EDL933. The study discovered host loci that facilitate EHEC infection of intestinal epithelial cells. The significant genes were associated with sphingolipid biosynthesis, particularly for the production of globotriaosylceramide (Gb3), the Stx receptor. Among all, two targeted loci (TM9SF2 and LAPTM4A) were novel. Knocking out these loci resulted in reduction of Stx-mediated cell death and EHEC T3SS-mediated cytotoxicity (Pacheco et al., 2018). More comprehensive studies on host genes interacting with virulent factors of EHEC are required to design host-directed therapies to control infection.

## Conclusion

This review describes the application of genome-wide CRISPR screens in infectious diseases to understand host pathogen interaction in depth. Establishment of infection is a multistep process and various host factors are involved in each step. CRISPR screens performed at genome wide level have contributed enormously to fundamental and translational research. Studying various host dependency factors in infectious diseases is necessary for discovery of targets for host directed therapies and to improve existing therapeutic strategies to combat infection in a better way. It is noteworthy that the reproducibility and accuracy of CRISPR screens is of high concordance. Minor disparities exist within the datasets obtained from independent CRISPR screens. This arises due to variations in experiment setup. However, most of the studies are in agreement when challenged with the same pathogens. Hence, this is the major advantage of using unbiased genome-wide CRISPR screens. The advent of combinatorial CRISPR screens will circumvent the subtle differences which are usually seen with normal screens. Thus, more comprehensive studies are required to understand host-pathogen interaction not only in the level of protein-protein but also in RNA-protein, RNA-RNA and DNA-protein interactions.
